# Integrated mechanistic and bioinformatics analysis of a traditional Chinese medicine compound MangHuang solution against *Candida albicans*

**DOI:** 10.3389/fcimb.2026.1737769

**Published:** 2026-03-27

**Authors:** Hansheng Zhu, Peijian Ma, Yefei Yuan, Yiping Chen, Hao Shi, Changyi Huang, Yue Zhou, Renzhi Jing, Mei Cao, Dan Zhang, Yu Luo

**Affiliations:** 1 School of Pharmacy, Southwest Medical University, Luzhou, China; 2 Key Laboratory of Biological Resource and Ecological Environment of Chinese Education Ministry, College of Life Sciences, Sichuan University, Chengdu, China; 3 R&D Department, Sichuan Golden Stone Hanfang Biotechnology Co., Ltd, Chengdu, China; 4 Core Laboratory, School of Medicine, Sichuan Provincial People’s Hospital Affiliated to University of Electronic Science and Technology of China, Chengdu, China

**Keywords:** antifungal mechanism, *Candida albicans*, MangHuang compound solution, multi-omics analysis, Traditional Chinese medicine

## Abstract

**Introduction:**

The growing prevalence of drug-resistant pathogens urgently calls for new treatment strategies. Traditional Chinese medicine (TCM) formulas, with their multi-targeted mechanisms of action, offer promising alternative options for antimicrobial therapy. This study aims to evaluate the antimicrobial activity of the TCM formula MangHuang solution (MH) after content detection of tannin, composed of *Rhei Radix et Rhizoma, Natrii Sulfas*, and *Galla Chinensis*, and to elucidate its antifungal mechanism against Candida albicans through integrated multi-omics analysis.

**Methods:**

MH and its individual or combined components were prepared and evaluated for their inhibitory effects on *Staphylococcus aureus, Escherichia coli*, and *Candida albicans*, and their antimicrobial activity was assessed. Transmission electron microscopy (TEM), biofilm formation experiments, and multi-omics analysis were used to investigate the antifungal mechanism of MH against *C. albicans*.

**Results:**

MH demonstrated potent and rapid antibacterial activity. Biofilm formation was significantly inhibited, manifested by reduced cell surface hydrophobicity, weakened initial adhesion capacity, and impaired biofilm maturation processes. Transcriptome and metabolome analyses revealed significant alterations in key metabolic pathways, particularly ABC transporters, amino acid biosynthesis, and protein-related pathways.

**Discussion:**

MH exhibits potent antifungal activity against *C. albicans* through a multi-target mechanism, primarily affecting biofilm formation and intracellular metabolic processes. The integration of multi-omics approaches provides strong evidence for the potential clinical application of MH as an effective antifungal agent.

## 1 Introduction

Environmental pollution and the widespread misuse of antibiotics have made microbial infections a critical global public health challenge (Holmes et al., 2016). A representative example is *Candida albicans*-induced vaginitis, which impairs patients’ quality of life, prolongs treatment, and elevates healthcare costs due to the pathogen’s increasing antibiotic resistance (Marnach et al., 2022). The conventional model for developing antibacterial drugs now faces significant obstacles, highlighting an urgent need to explore new strategies like anti-virulence and anti-biofilm approaches, as well as novel sources such as natural products.

*Candida albicans* represents a serious clinical threat, capable of causing invasive infections with a broad spectrum, high incidence, and significant mortality (Wasiński, 2019; Ahmad-Mansour et al., 2021). Its complex cell wall, rich in antigens and toxic compounds, facilitates evasion of immune responses and disruption of host homeostasis (Nobile and Johnson, 2015). Likewise, *Staphylococcus aureus* and *Escherichia coli* are major clinical pathogens; their numerous virulence factors, compounded by antibiotic overuse, greatly complicate treatment and pose an ongoing challenge to global public health (Ruiz-Herrera et al., 2006; Poulain, 2015). Together, these pathogens demonstrate enhanced adaptability, transmissibility, colonization capacity (Gulati and Nobile, 2016; Wall et al., 2019), and pronounced multidrug resistance (Pereira et al., 2021).

In Traditional Chinese Medicine (TCM) theory, pathogens are considered “evil qi” (xie qi), which are external pathogenic factors that can easily cause infection when the body’s vital functions are compromised (Methicillin-resistant staphylococcus aureus, 2018; Paitan, 2018). While TCM-based natural preparations are widely used, few single-ingredient agents possess potent antibacterial activity on their own; they typically require combination with antibiotics (Prasad et al., 2019) or are used as alternative therapies (Crowley and Gallagher, 2014).

MangHuang solution (MH) is a pure TCM formulation developed by a pharmaceutical company in Sichuan Province. It has been used clinically at institutions including Chengdu Anorectal Hospital, Henggang People’s Hospital of Shenzhen, and Sichuan Provincial Hospital of TCM for treating hemorrhoids and for perianal disinfection (Lu et al., 2021; Ren et al., 2021). Clinical observations confirm its notable antibacterial, anti-inflammatory, and wound-healing effects. The formulation comprises extracts from *Galla Chinensis* (Wubeizi)*, Rhei Radix et Rhizoma* (Dahuang), and *Natrii Sulfas* (Mangxiao). In TCM principles, these herbs are considered cold-natured and belong to the large intestine meridian, where they function to clear heat, purge fire, and remove toxins. Chemically, MH’s primary constituents are anthraquinones (from *Rhei Radix et Rhizoma*), sodium sulfate(from *Natrii Sulfas*), and tannin (from *Galla Chinensis*). These compounds demonstrate broad-spectrum antibacterial activity and can eliminate harmful metabolites and endotoxins produced during microbial infections (Xu et al., 2014; Xu et al., 2024).

This study investigates the antimicrobial activity of a TCM compound formulation, derived from the MH base, against *C. albicans* and other pathogens, following preliminary screening. We aim to elucidate its mechanisms of action to inform subsequent research and potential drug development.

## 2 Materials and methods

### 2.1 Preparation of Chinese herbal extracts

The traditional Chinese herbal compound solution MH consists of *Rhei Radix et Rhizoma* (30 g), *Natrii Sulfas* (30 g), and *Galla Chinensis* (30 g), prepared using the decoction method. The crude herbs were boiled in 600 mL of water over high heat, then simmered over low heat for 1 h. The extract is filtered and concentrated to obtain a solution with a total crude herbal material concentration of 0.3 g/mL. To investigate the contribution of individual herbal medicines and compound ratios, seven formulations (W–WDM) were designed, each maintaining a total crude herbal material concentration of 0.3 g/mL (Cheng et al., 2021; Yuan et al., 2024). The prescriptions for each formulations are presented in **Table 1**.

**Table 1:** The prescriptions of different formulations.

Group	Prescription
W	30 g *Galla Chinensis*, 200 mL water
D	30 g *Rhei Radix et Rhizoma*, 200 mL water
M	30 g *Natrii Sulfas*, 200 mL water
WD	30 g *Galla Chinensis*, 30 g *Rhei Radix et Rhizoma*, 400 mL water
WM	30 g *Galla Chinensis*, 30* g Natrii Sulfas*, 400 mL water
DM	30 g *Rhei Radix et Rhizoma*, 30 g *Natrii Sulfas*, 400 mL water
WDM (MH)	30 g *Galla Chinensis*, 30 g *Rhei Radix et Rhizoma*, 30 g *Natrii Sulfas*, 600 mL water

Accurately pipette 2.0 mL of the MH and evaporate it to dryness in a 50 °C water bath. The residue is then dissolved in 150 mL of water. The total tannin content is subsequently determined by preparing a test sample solution of the MH and establishing a tannin standard curve according to the method described above (Brêtas et al., 2020).

### 2.2 Antimicrobial activity determination

#### 2.2.1 Solid medium preparation and microbial strains

##### 2.2.1.1 Nutrient agar medium

It was prepared by dissolving the following components per liter: peptone, 10.0 g; beef extract, 3.0 g; sodium chloride, 5.0 g; and agar, 15.0 g. The pH was adjusted to 7.2 ± 0.2 using 1 M NaOH or HCl.

##### 2.2.1.2 Sabouraud dextrose agar medium

It was prepared by dissolving the following components per liter: dextrose, 40.0 g; peptone, 10.0 g; and agar, 15.0 g. The pH was naturally approximately 5.6 and was not further adjusted.

All media were prepared with deionized water and sterilized by autoclaving at 121°C for 15 min. The sterilized media were then poured into sterile petri dishes, with approximately 15–20 mL per plate, under aseptic conditions. The poured plates were allowed to solidify at room temperature and were either used immediately or stored at 4°C for up to two weeks.

*Staphylococcus aureus* (ATCC 6538) and *Escherichia coli* (ATCC 25922) were cultured on NA, while *Candida albicans* (ATCC 10231) was cultured on SDA. Commercial standard of *S. aureus*, *E. coli*, and *C. albicans* were purchased from BeNa Culture Collection and revived on the corresponding agar medium.

#### 2.2.2 Antimicrobial testing

Antimicrobial activity was assessed according to GB 15979–2002 and WS/T 650–2019 standards. The former is a Chinese national standard 15979-2002, while the latter is a Chinese health industry standard 650-2019. Both standards stipulate methods for evaluating the antibacterial activity of solutions and share similar experimental procedures. The practical protocol is described as follows.

Fresh 24 h cultures of *S. aureus* and *E. coli* on NA, and of *C. albicans* on SDA, were cultivated. The microbial cells were harvested by washing the agar surface with phosphate-buffered saline (PBS, pH 7.4, containing 137 mM NaCl, 2.7 mM KCl, 10 mM Na_2_HPO_4_, and 1.8 mM KH_2_PO_4_) to prepare the suspension.

Briefly, 0.1 mL bacterial or fungal suspension (~1×10^4^–9×10^4^ CFU/mL) were treated with 5 mL of each herbal medicine solution for 2, 5, 10, and 20 min. The reaction was terminated by dilution with 5mL PBS three consecutive times, and 0.5 mL aliquots from appropriate dilutions were plated. All plates were incubated at 36 ± 1 °C for 48 h for bacteria or 72 h for *C. albicans* prior to colony enumeration (relative humidity = 70%) (GB 15979-2002, 2002). All experiments were performed in triplicate (n = 3).

Bacteriostatic rate ≥ 50% - 90%, judged to have bacteriostatic effect; Bacteriostatic rate ≥ 90%, judged to have strong bacteriostatic effect. The bacteriostatic rate was calculated using the following formula:


$$\begin{array}{c} Bacteriostatic\;rate=\\ \frac{Average\;colony\;number\;of\;control-Average\;colony\;number\;of\;sample}{Average\;colony\;number\;of\;control}*100\% \end{array}$$

### 2.3 Mechanistic study of *Candida albicans*

#### 2.3.1 Growth curve analysis

Into pre-labeled 5 mL EP tubes, we first added 1 mL of a *C. albicans* suspension (OD_600nm_ = 0.4 in RPMI-1640 medium), followed by 1 mL of the drug solution from the MH. This yielded a final volume of 2 mL per tube, with final concentrations of 2.5 × 10^8^ CFU/mL for the fungus and 150 mg/mL for the drug. PBS served as the control. We monitored the *C. albicans* cultures in both the treated and untreated groups every 3 h over a 24 h period to construct growth curves (WS/T 650-2019, 2019; Su et al., 2021). PBS served as the control group, and all experiments were performed in triplicate (n = 3).

At the same time, fungistatic rate was used to calculate in section 2.2 to draw the Time-Kill curve.

#### 2.3.2 Transmission electron microscopy

The cultured condition was prepared in a growth medium containing either MH or PBS against *C. albicans*, following the procedure described in section 3.1. On the second day, fungal cells were harvested by centrifugation at 4000 rpm for 10 min. The supernatant was discarded, and the cell pellet was gently resuspended in 1 mL of 2.5% glutaraldehyde. The suspension was transferred to a 1.5 mL microcentrifuge tube and fixed by centrifugation at 10,000 rpm for 10 min. After carefully removing the supernatant, fresh 2.5% glutaraldehyde was slowly added along the tube wall to minimize disturbance to the cells. Samples were allowed to stand at room temperature for 30 min before being stored at 4°C (Tizro et al., 2019; Lu et al., 2023).

Subsequently, samples underwent dehydration, embedding, ultrathin sectioning, and staining. The sample rod was inserted to calibrate the optical path. The target area was first located in low-magnification mode, the sample position was adjusted, and imaging was then performed at high magnification. Morphological alterations of *C. albicans* were examined under a Hitachi HT-7800 TEM operated at 80 kV.

#### 2.3.3 Sorbitol protection assay

To determine whether the antifungal effect targets the cell wall, *C. albicans* was cultured in RPMI-1640 medium and RPMI-1640 medium containing 0.8 M sorbitol during treatment (Mishra and Wang, 2017; Mohammad Zadeh et al., 2021). PBS served as the control. Following a 24 h incubation at 37°C, the PBS was sequentially diluted 200-fold and then 10-fold. A 200 µL aliquot was plated onto SDA. Each group was plated in triplicate, and the average value was calculated. The fungistatic rate was calculated using the method described in section 3.1. PBS served as the control group, and all experiments were performed in triplicate (n = 3).

#### 2.3.4 Biofilm detection

Biofilm inhibitory activity was assessed using the following methods:

##### 2.3.4.1 Cell surface hydrophobicity (MATH assay)

The cultured environment was prepared according to the method in section 3.1, using RPMI-1640 medium without phenol red. Following a 24 h incubation at 37°C, a 100 µL aliquot from each group was transferred to a 96-well plate for measurement of the initial absorbance (A_0_) at 600 nm. Next, 1.5 mL of the suspension was combined with an equal volume of n-hexadecane, vortexed for 1 min, and incubated at room temperature for 15 min to achieve complete phase separation. The lower aqueous phase was carefully aspirated, and its absorbance (A_1_) was measured at 600 nm. The cell surface hydrophobicity was then calculated using the formula:


$$\text{C}ell\;surface\;hydrophobicity=\frac{A_{0}-A_{1}}{A_{1}}*100\%$$

##### 2.3.4.2 Initial adhesion

*C. albicans* was revived and cultured overnight on SDA. The following day, colonies were diluted in phenol red-free RPMI 1640 medium to prepare a fungal suspension with an OD_600nm_ at 0.4. A 100 μL aliquot of this suspension was added to each well of a 96-well plate containing 100 μL of different drug solutions. After incubation at 37°C for 2.5 h, all liquid was carefully aspirated from the wells. The wells were then washed twice with 200 μL of PBS to remove non-adherent fungi. Following PBS removal, 200 μL of methanol was added to each well for fixation for 15 min. The methanol was then aspirated, and the plate was air-dried in a biosafety cabinet. Subsequently, 200 μL of 1% crystal violet solution was added to each well for staining for 5 min. Excess stain was gently rinsed off under running water in a foam box until the water ran clear. The plate was again air-dried in the biosafety cabinet. Finally, 200 μL of 33% acetic acid was added to each well, and the OD_600nm_ (A_1_) was determined using a microplate reader.

The initial adhesion was then calculated using the Biofilm inhibitory activity was assessed using the following methods:


$$\text{Initial adhesion}=\frac{A_{1}-A_{control}}{A_{1}}*100\%$$

##### 2.3.4.3 Mature biofilm formation

The test method was consistent with section 3.2, and the incubation time was extended to 24 h on this basis (Zhang et al., 2011; Ma et al., 2023; Wang et al., 2024). PBS served as the control, and all experiments were performed in triplicate (n = 3).

#### 2.3.5 Morphological observation

The test method was consistent with formation of biofilm. Treated *C. albicans* cells were stained with Lugol’s iodine solution and observed under a light microscope to assess the conversion of yeast to hyphae. The test method followed the procedure outlined in section 3.4. Following the incubation period, images were captured using a microscope by 100x.

### 2.4 Transcriptome analysis

*C. albicans* cells treated with MH and gallic acid (70% purity, isolated from *Galla Chinensis* in the laboratory) were subjected to RNA extraction, quality control (RIN > 8.0), and cDNA library construction (Lu et al., 2023). The library was sequenced on the Illumina platform. Differentially expressed genes (DEGs) were identified using a threshold of |log_2_FC| ≥ 1 and FDR< 0.05, followed by Gene Ontology (GO) and Kyoto Encyclopedia of Genes and Genomes (KEGG) enrichment analysis.

### 2.5 Metabolomics analysis

Fresh 24 h slant cultures of the test strain (*C. albicans*) were washed off with sterile PBS. The cell suspension was adjusted to an OD_600nm_ of 0.4 using PBS as determined by a microplate reader. Drug A was prepared in PBS at 100 mg/mL, and Drug B was dissolved in PBS to 60 mg/mL. In a sterile 10 mL centrifuge tube, 4.0 mL of the corresponding drug solution was added first and pre-equilibrated in a 20 ± 1°C water bath for 5 min. Subsequently, 4.0 mL of the prepared fungal suspension was added, and the mixture was immediately vortexed to ensure thorough mixing. The cultures were incubated for 24 h. After incubation, samples from each group were collected; the cultured supernatants were harvested and stored at −80°C until further metabolomics analysis. Metabolite profiling was performed using ultra-high-performance liquid chromatography-mass spectrometry (UHPLC-MS). Differentially expressed metabolites (VIP > 1.0, p< 0.05) were identified using multivariate statistical analysis (PCA, OPLS-DA), followed by KEGG pathway annotation.

### 2.6 Integrated multi-omics analysis

Transcriptomics and metabolomics datasets from matched samples were integrated to investigate coordinated molecular changes. Normalized gene expression and metabolite abundance matrices were used to define differentially expressed genes (DEGs) and differentially expressed metabolites, respectively. Pearson’s correlation coefficients were then calculated across samples to quantify DEG–metabolite associations, and statistically significant correlations (with multiple-testing correction where applicable) were retained for interpretation. To contextualize these cross-omics relationships, DEGs and differential metabolites were mapped to KEGG and subjected to pathway analysis, and pathways supported by both omics layers were identified as overlapping regulatory effects.

### 2.7 Statistics

PBS was used as the control group, and all experiments were performed in triplicate (n = 3). Data are expressed as mean ± standard deviation (SD). Significant differences were assessed using one-way or two-way analysis of variance (ANOVA) on GraphPad Prism 9, version 9.5.1.

## 3 Results

### 3.1 Determination of tannin content in MangHuang solution

**Figure 1:**
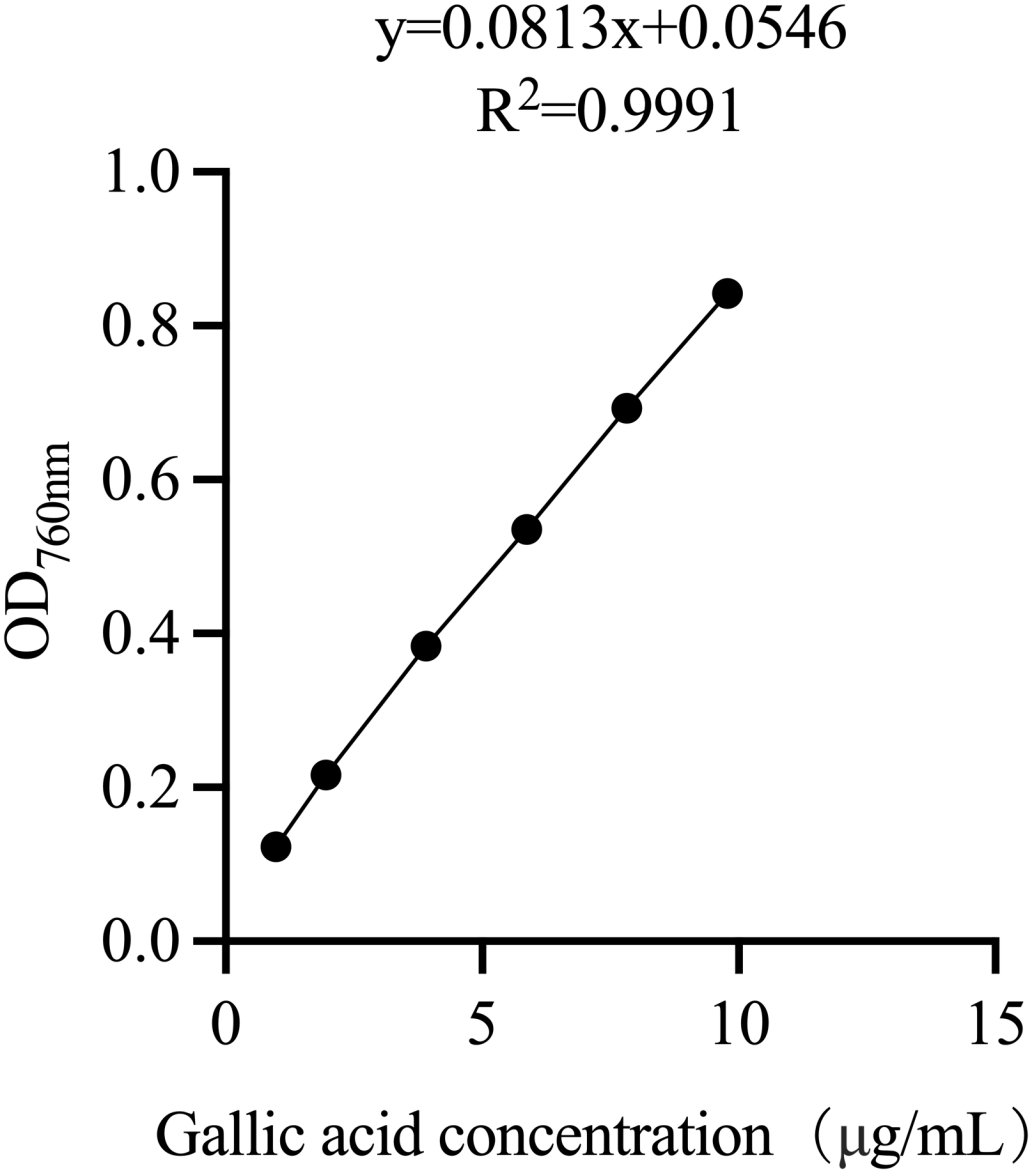
Standard curve of gallic acid. y = 0.0813 x + 0.0546, R^2^ = 0.9991.

**Figure 1** presents the standard curve for gallic acid. The relationship between concentration and absorbance is linear, with a regression equation of y = 0.0813x + 0.0546 and an R² value of 0.9991. This indicates a strong correlation.

The analysis yielded an average total tannin concentration of 0.766 mg/mL across six batches of MH, with a relative standard deviation (RSD) of 2.377%. The detailed calculations and results are provided in **Table 2**.

**Table 2:** Measured and calculated values of tannin content for 6 Batches of MH (n = 6).

Batch number	F1122	F1123	F1124	F1125	F1126	F1127
A _Total phenols_	0.472	0.479	0.466	0.451	0.439	0.442
A _Non absorbed polyphenols_	0.196	0.196	0.168	0.179	0.168	0.165
A _Tannin content_	0.250	0.257	0.246	0.246	0.245	0.251
Tannin content (mg/mL)	0.769	0.797	0.753	0.753	0.749	0.773

A _Tannin content_ = A _Total phenols-A Tannin content_-0.026, while 0.026 represents the adsorption value of casein; Tannin content = (A _Tannin content_ - 0.0546)/0.0813 * 0.32, 0.32 was the dilution factor in actual process.

### 3.2 Antimicrobial activities

**Figure 2:**
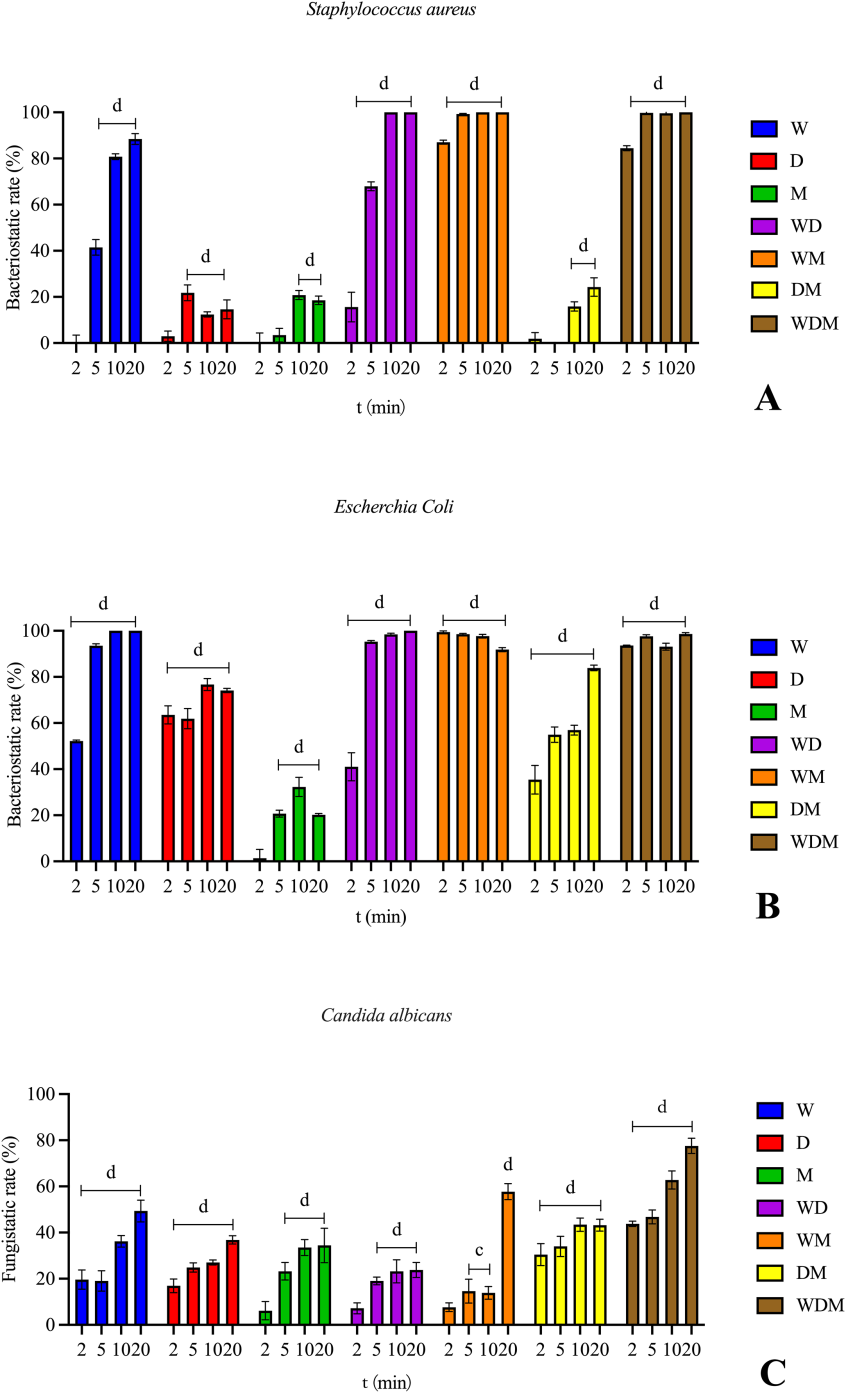
Antimicrobial activity of MH. **(A)** (S) aureus. **(B)***E. coil*. **(C)** albicans. (W) Galla chinensis. (D) Rhei Radix et Rhizoma. (M) Natrii Sulfas. (WD) Galla chinensis and Rhei Radix et Rhizoma. (WM) Galla chinensis and Natrii Sulfas. (DM) Rhei Radix et Rhizoma and Natrii Sulfas. (WDM) Galla chinensis, Rhei Radix et Rhizoma and Natrii Sulfas. The values represent the mean ± SD (n=3). A indicates a significant difference compared to the control group (p < 0.05), b indicates p < 0.01, c indicates p < 0.001, and d indicates p < 0.0001.

The W, WD, WM, and WDM groups exhibited potent antimicrobial activity against both Gram-positive (*S. aureus*) and Gram-negative (*E. coli*) bacteria, achieving bacteriostatic rates exceeding 90% within 20 min. Notably, the WM and WDM groups, particularly the latter (MH), demonstrated rapid bactericidal activity, reducing viable bacterial counts by over 90% within just 5 min. Compared to the D and M groups, the W group showed superior efficacy, suggesting that *Galla chinensis* contributed more prominently to the observed antimicrobial activity than *Rhei Radix et Rhizoma* and *Natrii Sulfas* (**Figure 2A**).

For *E. coli*, the D group exhibited stronger inhibition than for *S. aureus*, resulting in the DM group being more effective against *E. coli*. At 20 min, the bacteriostatic activity of the DM group against *E. coli* increased by approximately 10% compared to the D group alone, indicating a synergistic effect of combining *Rhei Radix et Rhizoma* and *Natrii Sulfas* without altering the total crude drug concentration (**Figure 2B**).

Regarding antifungal activity, significant inhibition was observed only in the WM and WDM groups, with fungistatic rates exceeding 50% within 20 min. Remarkably, the WDM group (MH) displayed earlier efficacy, achieving noticeable inhibition at 10 min and exhibiting approximately 20% higher fungistatic activity than the WM group at 20 min (**Figure 2C**).

### 3.3 Growth kinetics of *Candida albicans* in response to MangHuang solution treatment

**Figure 3:**
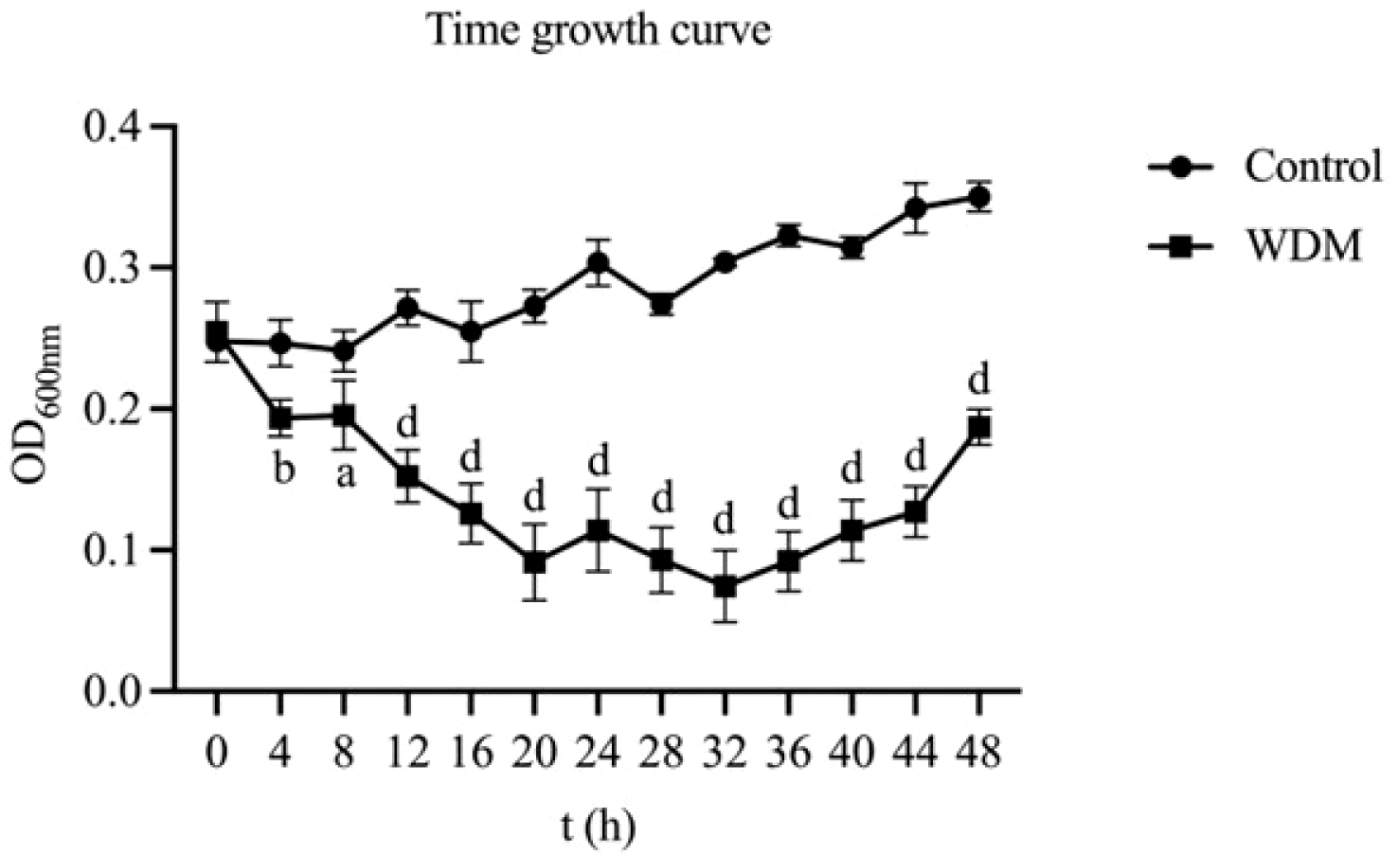
Time growth curve of MH on *C. albicans* (n = 3). The values represent the mean ± SD (n=3). A indicates a significant difference compared to the control group (*p* < 0.05), *b* indicates *p* < 0.01, *c* indicates *p* < 0.001, and d indicates *p* < 0.0001.

As shown in **Figure 3**, *C. albicans* treated with the WDM group (MH) exhibited a markedly slower growth pattern, with cell viability progressively declining from 0 to 32 h. A significant reduction in *C. albicans* concentration was observed between 4 and 48 h (p< 0.05). In contrast, the control group displayed a typical growth curve: a lag phase during the first 8 h, followed by a steady increase in absorbance indicative of the logarithmic phase.

These findings suggest that the MH inhibits *C. albicans* growth primarily by suppressing its reproductive capacity during both the lag and logarithmic phases. Notably, the fungistatic rate at 24 h aligns with the results of **Figure 2C**, supporting the selection of a 24-h treatment duration for subsequent assays.

**Figure 4:**
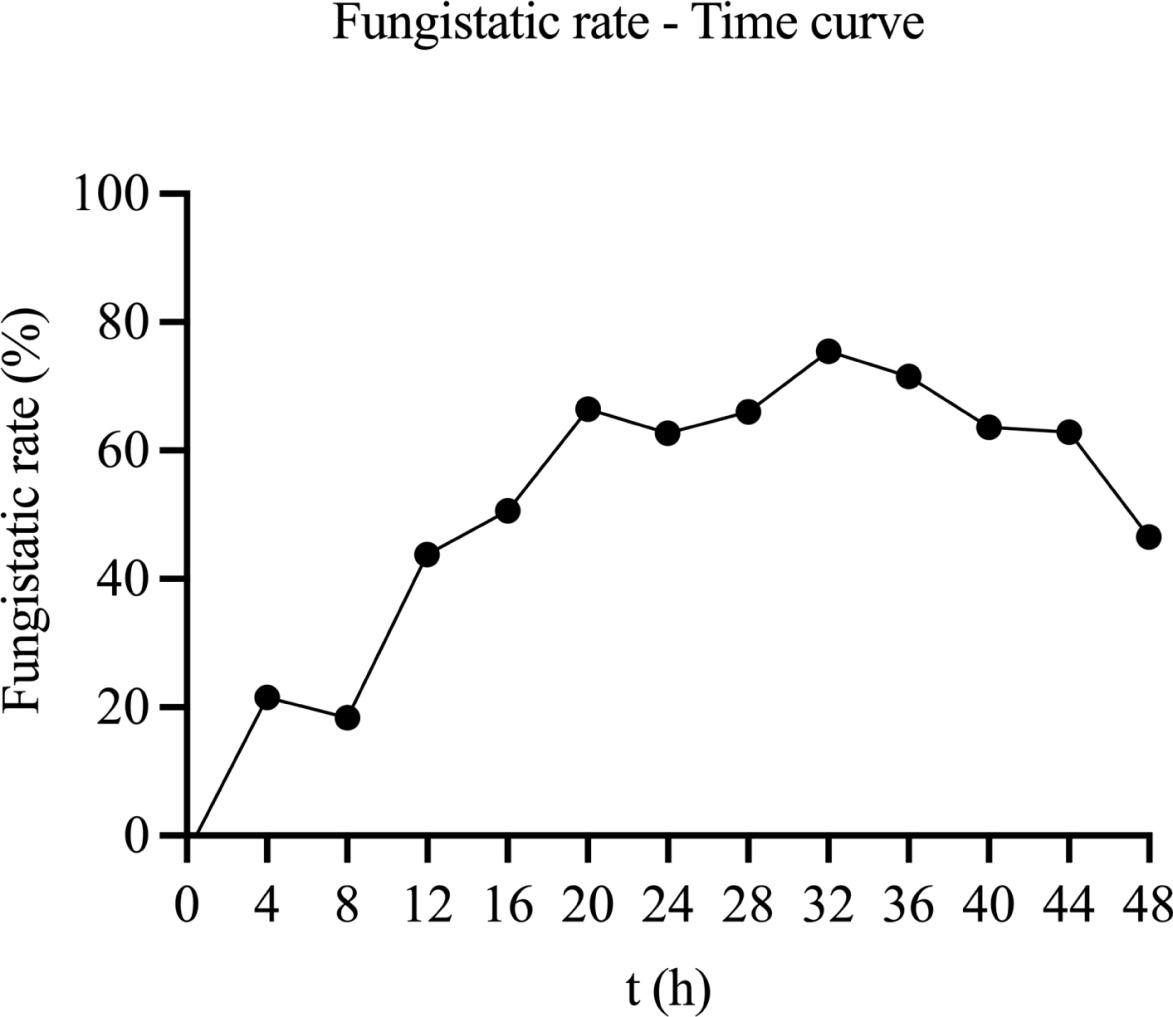
Fungistatic rate - Time curve of MH against *C. albicans*.

As illustrated in **Figure 4**, the fungistatic activity of MH reached its maximum at 32 h post-treatment, indicating the most effective inhibition of *C. albicans* at this time. A notable increase in activity was also detected at 20 h, providing a rationale for the 24-h dosing interval. Beyond 32 h, the inhibitory effect of the formulation slowly declined.

### 3.4 Observation of *Candida albicans* ultrastructure by TEM

**Figure 5:**
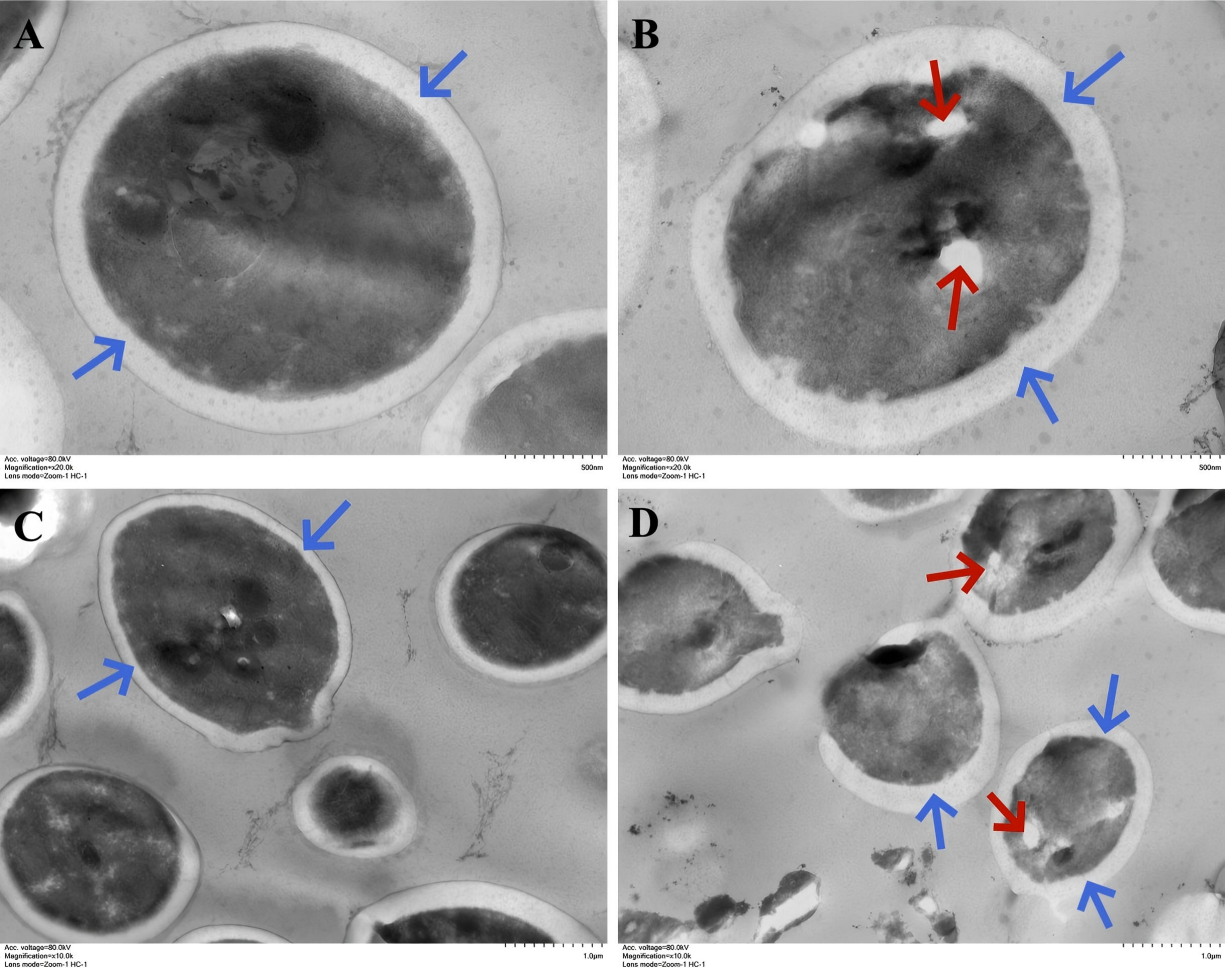
Ultrastructural features of observed by TEM. **(A)** Single-cell image of *C. albicans* in the control group. **(B)** Single-cell image of in the experimental group. **(C)** Multi-cell image of in the control group. **(D)** Multi-cell image of in the experimental group. **(A, B)** Scale bar is 500 nm. **(C, D)** Scale bar is 1 μm.

As shown in **Figure 5**, the cell walls of *C. albicans* in both the control and treatment groups remained largely intact (blue arrows). However, in cells treated with the WDM group (MH), the cytoplasm appeared heterogeneous, with regions of high electron-density aggregation, vacuolization, and bright cytoplasmic areas (red arrows). These ultrastructural alterations indicate that the antimicrobial mechanism of MH is unlikely to involve direct disruption of the cell wall. Instead, it may be associated with effects on intracellular processes, including gene expression, protein integrity, and metabolic activity.

**Figure 6:**
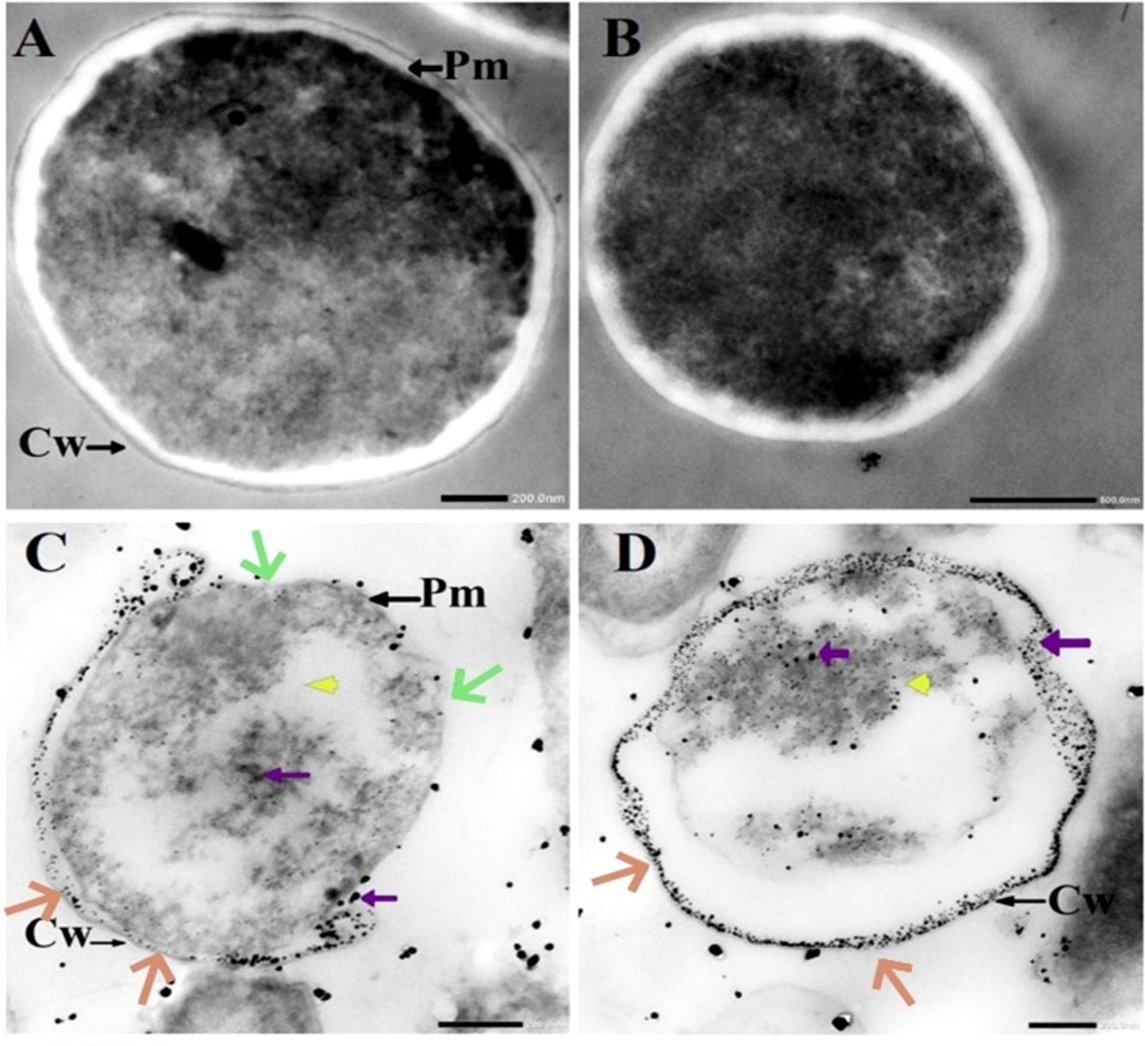
Ultrastructural features of *C. albicans* cell wall disruption by TEM. **(A, B)** Micrographs of untreated *C. albicans* exhibiting an intact cell wall (Cw) and intact plasma membranes (Pm). **(B)** Single-cell image of *C.* in the experimental group. **(C, D)** Micrographs of *C. albicans* treated with 1.5 mg/mL AgNO_3_ showing the disruption. Reprinted from (Hamida et al., 2021). Scale bar is 200 nm.

As positive controls, we cite TEM images of untreated *C. albicans* cell walls and those treated with 1.5 mg/mL AgNO_3_, which reveal clear structural disruption (Hamida et al., 2021). Silver ions are known to exert potent inhibitory and lethal effects on pathogenic microorganisms. Accordingly, images **Figures 6C, D** show not only cytoplasmic lysis (yellow arrows) but also a marked reduction in cell wall thickness relative to **Figures 6A, B**; the white peripheral region has thinned to near invisibility (brown arrows). In **Figure 6C** particularly, more than half of the upper cell wall appears dissolved (green arrows). The purple arrows indicate metal particles synthesized by the *C. albicans* cells themselves.

### 3.5 Verification of cell wall integrity by sorbitol

**Figure 7:**
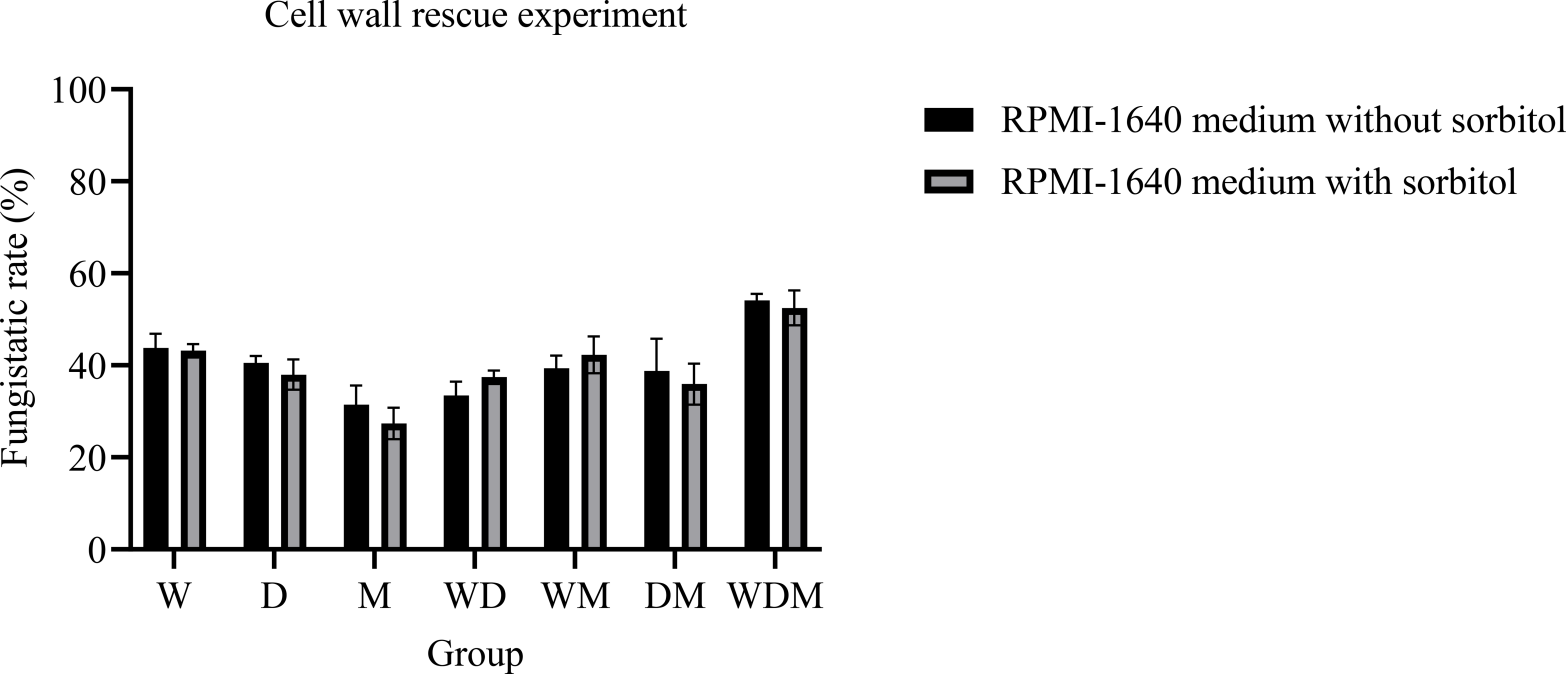
Cell wall rescue experiment (n=3). (Black column) RPMI-1640 medium without 0.8 M sorbitol. (Gary column) RPMI-1640 medium contain 0.8 M sorbitol.

The trends from the antimicrobial activity tests were consistent with the antifungal effects of the experimental drug solutions. Even with the protective agent sorbitol, the damage to *C. albicans* remained comparable to that observed without protection. Specifically, the inhibition rate on the sorbitol-containing specialized medium showed no significant difference from that on the conventional medium. TEM imaging further suggests that the antifungal effects of the seven herbal combinations are not primarily mediated through the fungal cell wall. Any cell wall damage appears minimal and unlikely to substantially impair the growth and reproduction of *C. albicans* (**Figure 7**).

### 3.6 Effect of MangHuang solution and its decomposed prescriptions on *Candida albicans* biofilm

#### 3.6.1 CSH, initial adhesion, and biofilm formation

**Figure 8:**
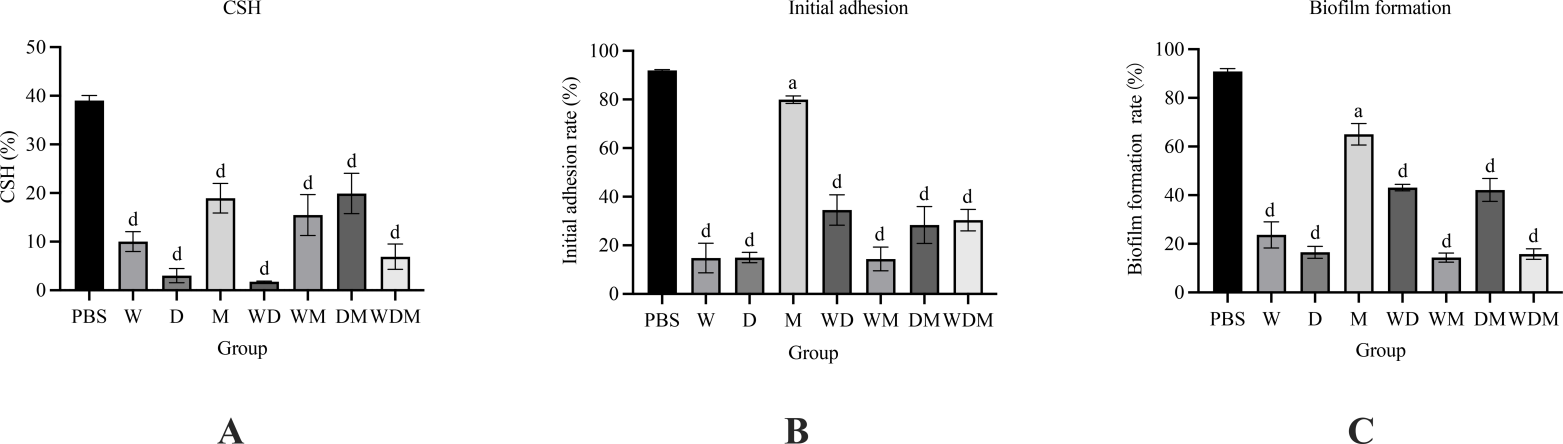
Effect of MH and its decomposed prescriptions on *C. albicans* biofilm (n = 3). **(A)** CSH. **(B)** Initial adhesion. **(C)** Biofilm formation. The values represent the mean ± SD (n=3). A indicates a significant difference compared to the control group (*p* < 0.05), *b* indicates *p* < 0.01, *c* indicates *p* < 0.001, and d indicates *p* < 0.0001.

This study first examined the effect of MH and its decomposed prescriptions on the cell surface hydrophobicity (CSH) of *C. albicans*. As shown in **Figure 8A**, the CSH of the control group was the highest at 39.03 ± 1.03%. After 24 h of treatment, CSH was reduced across all seven experimental groups. Notably, the D group, WD group, and WDM group (MH) exhibited the most significant reductions, with CSH values dropping below 10%: 3.01 ± 1.46%, 1.72 ± 0.15%, and 6.90 ± 2.60%, respectively. These three groups contained *Rhei Radix et Rhizoma*, suggesting a potential role in inhibiting *C. albicans* CSH. The combined use of *Rhei Radix et Rhizoma* and *Galla Chinensis* appeared to enhance this inhibitory effect.

Reduced CSH likely impaired the ability of *C. albicans* to overcome environmental electrostatic repulsion, thereby hindering adhesion to host surfaces. Consequently, initial adhesion rates were also affected. In the control group, the initial adhesion rate was 91.96 ± 0.31%. The W, D, and WM groups showed the largest decreases, while the WDM group, WD group, and DM group exhibited adhesion rates of 30.39 ± 4.41%, 34.54 ± 6.23%, and 28.38 ± 7.57%, respectively. The M group demonstrated the smallest reduction (p< 0.05), whereas differences in the other groups were highly significant (p< 0.0001) (**Figure 8B**).

The biofilm formation rate at 24 h in untreated *C. albicans* was 90.87 ± 1.18%. Overall, trends in biofilm formation mirrored those of initial adhesion. The W, D, and WM groups maintained low levels of biofilm formation, with the WDM group ranking second among all experimental groups, showing a biofilm formation rate of 15.80 ± 2.18% (**Figure 8C**).

The absorbance values for CSH, initial adhesion, and biofilm formation in the presence of n-hexadecane or 33% acetic acid are also shown (A_0_ and A_1_, **Table 3**). A smaller difference between A_0_ and A_1_ indicates greater damage to the *C. albicans* biofilm caused by the decomposed formulation. All data represent the mean of three independent replicates (x̄ ± SD, n=3).

**Table 3:** Detailed absorbance values in biofilm experiments (x̄ ± SD).

Group	CSH		Initial adhesion		Biofilm formation	
	A_0_	A_1_	A_0_	A_1_	A_0_	A_1_
PBS	0.551 ± 0.026	0.336 ± 0.021	1.027 ± 0.025	0.083 ± 0.002	1.088 ± 0.120	0.098 ± 0.002
W	1.163 ± 0.018	1.047 ± 0.040	0.364 ± 0.011	0.310 ± 0.014	0.519 ± 0.056	0.397 ± 0.065
D	1.470 ± 0.022	1.425 ± 0.007	0.496 ± 0.021	0.421 ± 0.016	0.301 ± 0.041	0.251 ± 0.027
M	0.543 ± 0.040	0.439 ± 0.017	0.493 ± 0.018	0.099 ± 0.009	0.537 ± 0.072	0.186 ± 0.009
WD	1.218 ± 0.001	1.197 ± 0.001	0.375 ± 0.032	0.244 ± 0.002	0.295 ± 0.018	0.168 ± 0.007
WM	1.070 ± 0.031	0.905 ± 0.071	0.194 ± 0.020	0.166 ± 0.026	0.199 ± 0.006	0.171 ± 0.004
DM	1.078 ± 0.080	0.863 ± 0.076	0.340 ± 0.040	0.242 ± 0.002	0.872 + 0.026	0.504 ± 0.039
WDM	1.270 ± 0.027	1.182 ± 0.008	0.256 ± 0.023	0.178 ± 0.024	0.231 ± 0.014	0.195 ± 0.007

#### 3.6.2 Qualitative analysis

**Figure 9:**
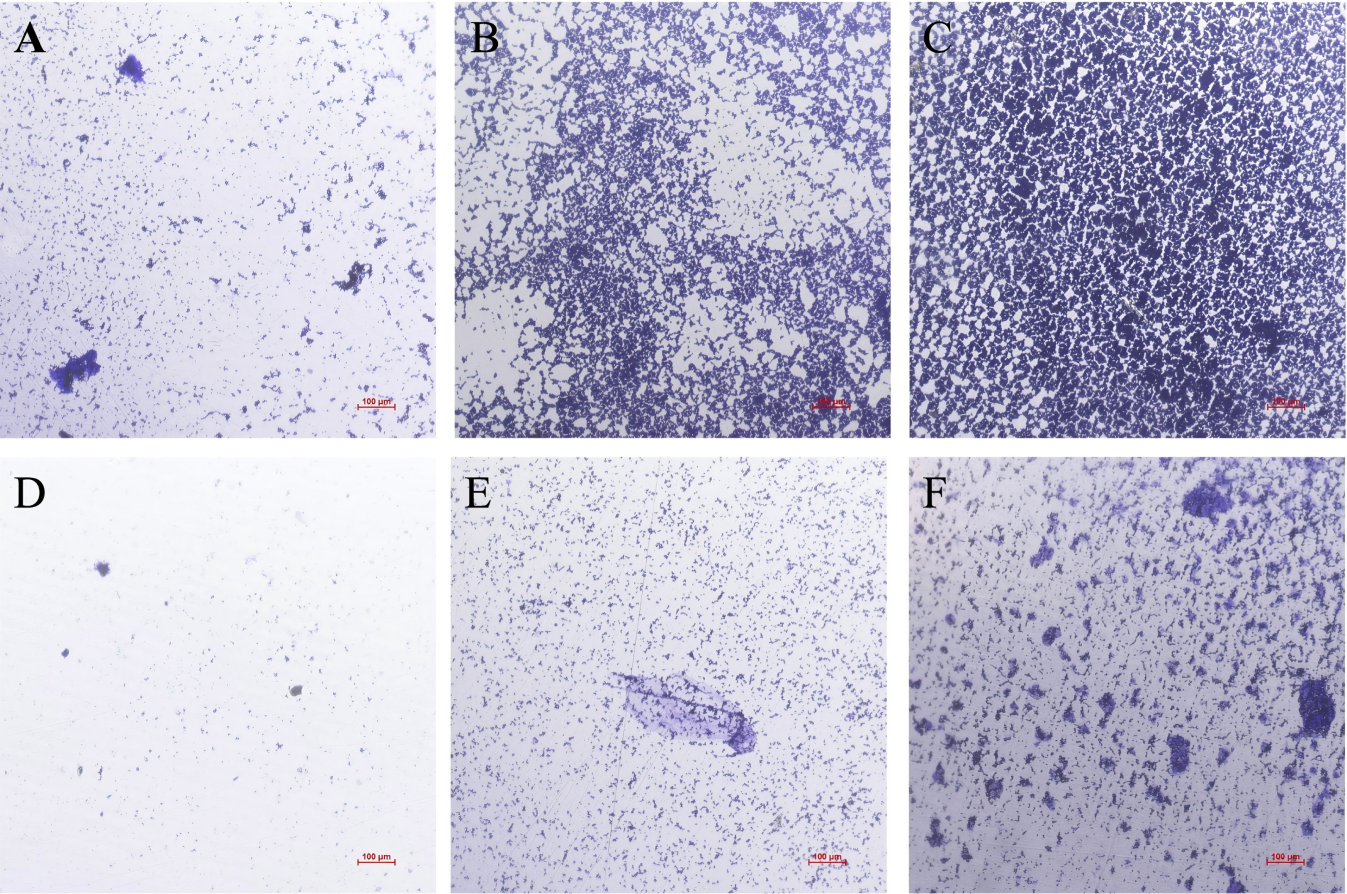
Qualitative analysis of *C. albicans* biofilm between control group and WDM group at 2.5, 12, 24 h. **(A)** Biofilm image of *C. albicans* in the control group at 2.5 h. **(B)** Biofilm image of *C. albicans* in the control group at 12 h. **(C)** Biofilm image of *C. albicans* in the control group at 24 h. **(D)** Biofilm image of *C. albicans* in the WDM group at 2.5 h. **(E)** Biofilm image of *C. albicans* in the WDM group at 12 h. **(F)** Biofilm image of *C. albicans* in the WDM group at 24 h. Scale bar is 100 μm.

Inverted microscopy revealed a substantial presence of budding cells in the control group at 2.5 h. These cells, appearing as short and granular under crystal violet staining, represented pseudohyphae of *C. albicans*. At 12 h, hyphal elongation became pronounced, with cells and hyphae aggregating to form clusters. By 24 h, the entire field of view was covered by cells and hyphae, forming a dense, intertwined network. These observations confirmed the successful establishment of a biofilm model in this experiment. The biofilm architecture at all time points in the control group was particularly prominent following crystal violet staining (**Figure 9**).

In contrast, *C. albicans* treated with the MH exhibited markedly slower biofilm development. Even at the end of the treatment period, the fungal cells failed to establish a dense biofilm structure.

### 3.7 Transcriptomic analysis of the mechanism by which MangHuang solution inhibits *Candida albicans* growth

To elucidate the mechanism underlying the inhibitory effect of MH on *C. albicans*, RNA-Seq was performed on samples from both untreated controls and *C. albicans* treated with MH. After quality control and removal of low-quality reads, an average of at least 20 million reads per sample was obtained, yielding a total of 54.96 Gb of clean data.

**Figure 10:**
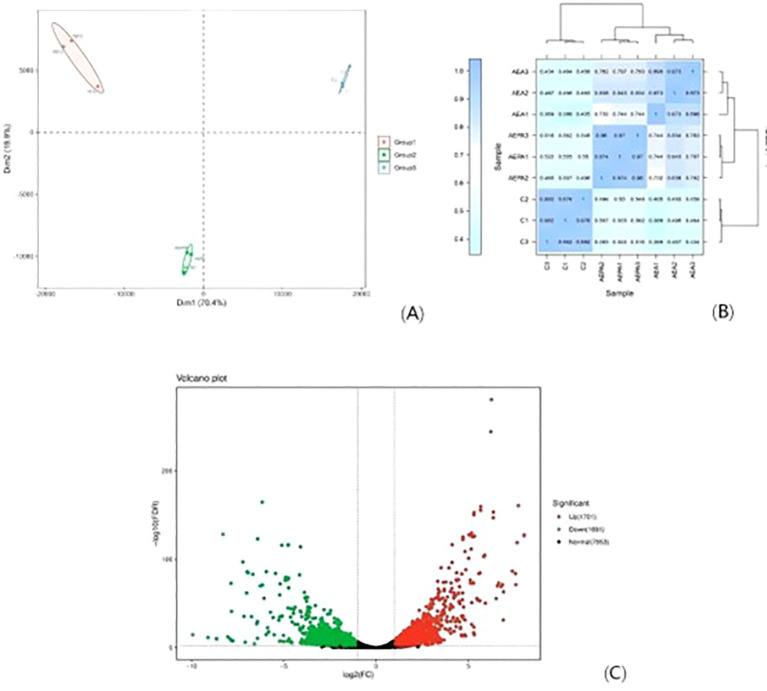
Transcriptome analysis of Muang on *C. albicans*. **(A)** PCA results for each group. Group 1 (MH + *C. albicans*), Group 2 (70% gallic acid + *C. albicans*) and Group 3 (PBS + *C. albicans*) **(B)** Heatmap of correlation between samples. **(C)** Volcanic plot of Control group vs WDM group in DEGs.

Comparative genome analysis between the control and WDM groups (MH) was conducted. PCA results indicate significant differences among the groups (**Figure 10A**). The experimental design was validated by assessing the correlation among biological replicates, as shown in the correlation heatmap (**Figure 10B**), which confirmed high reproducibility across samples. Differentially expressed genes (DEGs, Fold Change≥2 and FDR<0.01) were visualized using a volcano plot, revealing a total of 3,392 DEGs, comprising 1,701 upregulated and 1,691 downregulated genes (**Figure 10C**).

### 3.8 GO enrichment and KEGG pathway analysis

**Figure 11:**
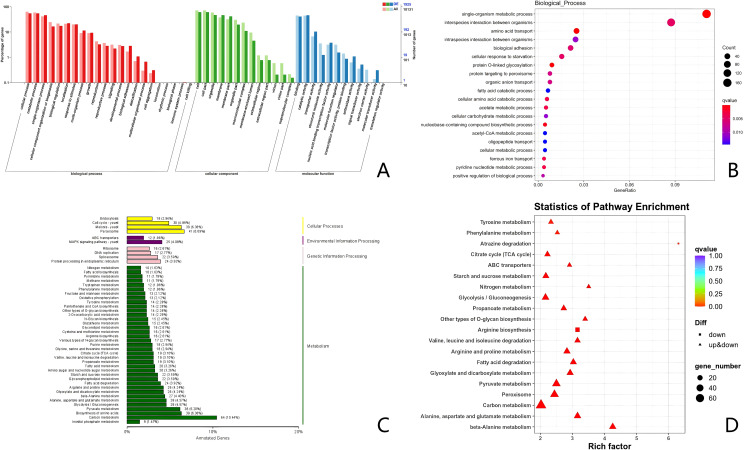
GO enrichment and KEGG pathway analysis of MH on *C. albicans*. **(A)** Go classification of Control group vs WDM group. **(B)** Biological process enrich of Control group vs WDM group. **(C)** KEGG enrichment of Control group vs WDM group. **(D)** KEGG classification of Control group vs WDM group.

GO annotation analysis was conducted on all DEGs to explain the interference effect of MH from three perspectives: biological process, cellular component, and molecular function. The results indicated that the membrane and organelles in *C. albicans* were altered, which affected its cellular processes. Furthermore, significant changes were observed in cellular and metabolic processes within the biological processes category (**Figure 11A**).

KEGG pathway analysis of the DEGs (**Figure 11C**) revealed that MH significantly impacted carbon metabolism, pyruvate metabolism, and amino acid biosynthesis in *C. albicans*. These processes are critical for maintaining the growth and viability of *C. albicans*. Additionally, MH also affected various biological modules in *C. albicans*, including the proteasome, MAPK signaling pathway, and meiosis.

Through further GO enrichment analysis of biological processes (**Figure 11B**), it was found that MH mainly interferes with the single biological metabolic process and the response to oxidative stress in *C. albicans*. KEGG pathway enrichment analysis (**Figure 11D**) revealed that, under the influence of MH, the most significant DEGs were enriched in carbon metabolism and the biosynthesis of cofactors in *C. albicans*. Therefore, the results of both GO annotation analysis and KEGG pathway annotation point to the involvement of material metabolism and cell structure functions.

### 3.9 Validation of MangHuang solution effects on *Candida albicans* biofilm-related gene expression by RT-qPCR

Based on previous transcriptomic analysis and literature reports (Lu et al., 2023), seven DEGs critically associated with *C. albicans* biofilm formation were selected for validation. Primer information for all relevant genes has been shown in **Table 4**. These genes—ALS1, ALS2, ALS4, ALS6, ECE1, CHT2, and ROB1—were prioritized due to their established roles in key biofilm processes: the ALS gene family mediates fungal adhesion to host surfaces; ECE1 is crucial for hyphal growth and invasion; CHT2 and ROB1 are involved in cell wall integrity and stress response, all of which are central to the biofilm phenotype observed in our study. This study aimed to investigate the roles of these key DEGs in biofilm development and to elucidate how MH modulates biofilm formation through their regulation. The primers and reference genes used for validation are detailed in the following methods section.

**Table 4:** Primer information of related genes.

Gene name	Sequence (5′ - 3′)	Product size (bp)
18s rRNA	F - GCA GTG GCA TCT CTC AGT C R - TCA TCG ATG CCA GAA CC	128
ALS1	F - TTA CTG CTC CTC CAG GTG GT R - CCG TGT AGT TTG GTG GCT CT	168
ALS2	F - CCA CAA CTA CCG TGA CTG CT R - AAC GGT ATC AGT GCC ACC TG	152
ALS4	F - CAC TGT TAC TGC CCC TCC AG R - AGC AGT CAC GGT AGT TGT GG	121
ALS6	F - AGA GCG TTT GGT ACC GTC AG R - ACA GTG TTC GTT CCA GCA GT	107
ECE1	F - ATT GTT GCT CGT GTT GCC AC R - CCA GGA CGC CAT CAA AAA CG	133
CHT2	F - TGC CAC CAC TAC TTC TGC TG R - GTG GTG GCA GCA TTT TCG TT	168
ROB1	F - CAC CTC CAC CAC TGT TAC CA R - TTG ATT TCC CAC CAG GTG CA	129

Specifically, ALS1, ALS2, and ALS4 act as positive regulators critical for the initial attachment and adhesion of yeast/planktonic cells to host surfaces. ECE1 and CHT2 are involved in the initiation of early-stage biofilm formation, while ROB1 functions as a master regulator facilitating hyphal development within the extracellular matrix (ECM).

**Figure 12:**
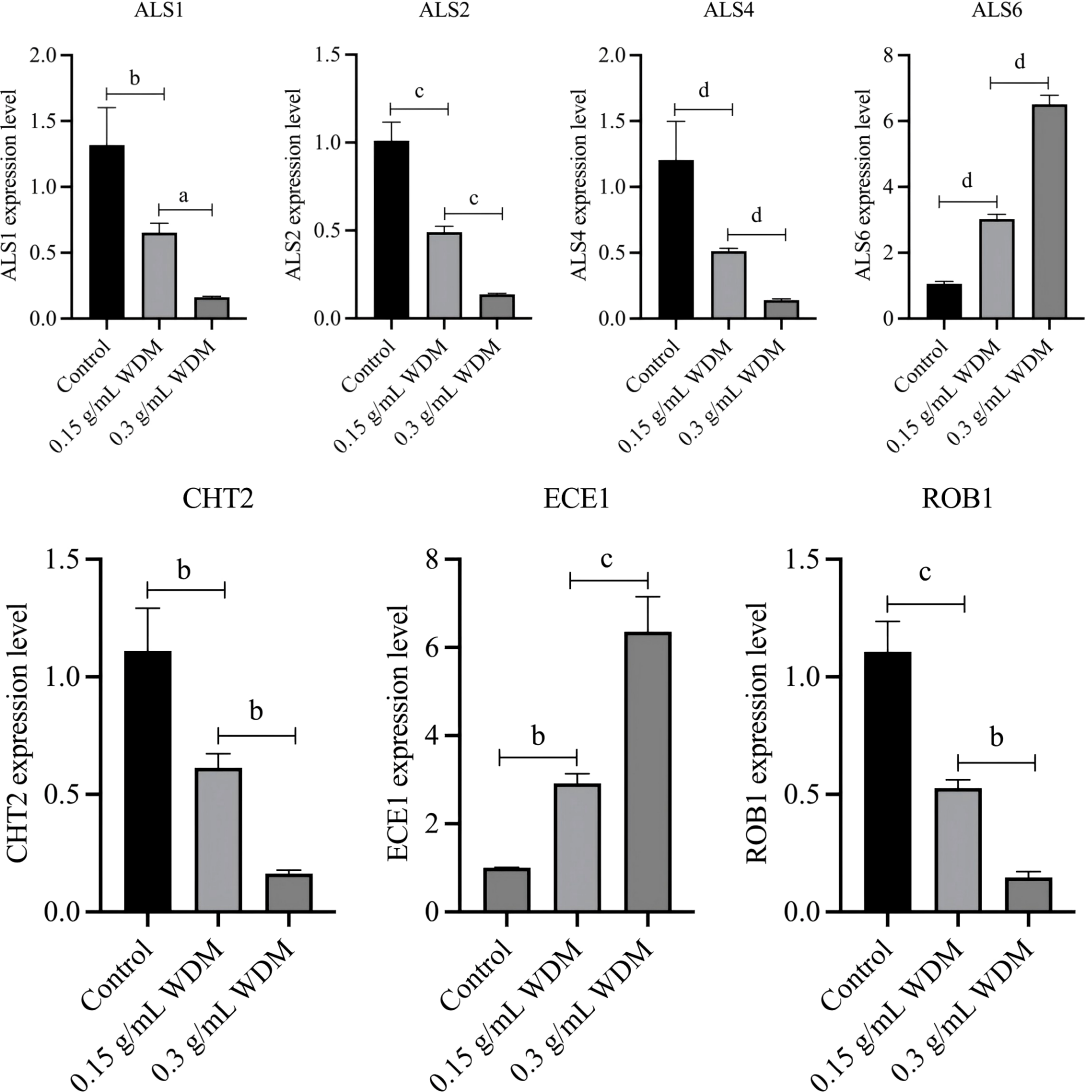
RT-qPCR verification of biofilm-related DEGs showing differential regulation. The values are expressed as the mean ± SD (n=3). A indicates a significant difference between the two groups (*p* < 0.05), b indicates *p* < 0.01, c indicates *p* < 0.001, and d indicates *p* < 0.0001.

To explore these mechanisms, KEGG pathway enrichment analysis was performed to identify biofilm-related genes with high expression abundance and significant roles in membrane-associated functions. RT-qPCR results demonstrated that treatment with MH at concentrations of 0.15 g/mL and 0.3 g/mL led to significant alterations in gene expression levels (**Figure 12**). The magnitude of these changes increased with higher concentrations of the crude drug.

Comprehensive analysis of gene expression profiles revealed that MH differentially regulates biofilm-associated genes. Notably, expression of positive biofilm regulators such as ALS1 and ALS2 was significantly downregulated, whereas genes potentially involved in anti-adhesive mechanisms, such as ALS6 (Zhao et al., 2007), were upregulated. These findings suggest that MH inhibits *C. albicans* growth by disrupting biofilm formation pathways (**Figure 10**). Functional annotations for these genes were retrieved from the NCBI Gene database.

## 4 Discussion

The results demonstrated significant broad-spectrum antimicrobial activity of the WDM group (MH) against *S. aureus*, *E. coli*, and *C. albicans*. These rapid effects suggest that MH may act independently of the concentration-dependent pharmacodynamics observed in conventional antibiotics. For instance, aminoglycosides inhibit bacterial protein synthesis in a concentration-dependent manner; however, their clinical utility is limited by dose-related ototoxicity and nephrotoxicity due to high residual concentrations at extended dosing intervals (Eyler and Shvets, 2019).

In contrast, traditional Chinese medicine (TCM) and its active constituents exhibit antibacterial effects against antibiotic-resistant pathogens, enhancing bacterial susceptibility to conventional antibiotics while reducing both the required dosage and the emergence of resistance (Li et al., 2022). MH may contribute to mitigating antimicrobial resistance due to its multi-component, multi-target, and multi-pathway characteristics, which make it challenging for pathogens to develop resistance against all pharmacodynamic mechanisms simultaneously (Zou et al., 2023). Moreover, certain TCM compounds have been shown to eliminate resistance genes and plasmids, thereby maximizing the efficacy of bacteriostatic agents (Liu et al., 2022). This approach also aligns with sustainable resource use and promotes a green economy.

The rapid bacteriostatic effect of MH is likely attributable to synergistic actions among its components. *Galla Chinensis* is rich in gallnut tannins, which disrupt biofilm architecture, interfere with bacterial growth and metabolism, and inhibit biofilm formation (Keyvani-Ghamsari et al., 2023). Similarly, anthraquinones in *Rhei Radix et Rhizoma* exert antimicrobial effects by impeding biofilm development, inhibiting toxin production, and disrupting nucleic acid and protein synthesis, ultimately blocking bacterial energy metabolism (Qun et al., 2023). Additionally, *Natrii Sulfas* contributes anti-inflammatory activity and scavenges harmful substances such as oxygen free radicals and inflammatory mediators (Xu et al., 2014). However, its combination with *Rhei Radix et Rhizoma* decoctions may reduce levels of certain active compounds (Doui et al., 2010).

The WDM group demonstrated stronger antibacterial activity than antifungal activity, possibly due to the structural differences of *C. albicans*. The fungal cell wall, composed predominantly of glucose, chitin, and mannose, serves as a protective barrier that limits the penetration and efficacy of TCM preparations (Chaffin et al., 1998; Childers et al., 2020). This structural feature may explain the relatively reduced susceptibility of *C. albicans* compared to bacteria.

Biofilm formation in *C. albicans* begins with changes in cell surface hydrophobicity (CSH), a key determinant of fungal interactions with external environments (Goswami et al., 2017). Pathogenic fungi typically exhibit high CSH, facilitating adhesion and biofilm formation (Rosenberg, 2017; Keyvani-Ghamsari et al., 2023). The biofilm developmental process includes four stages: surface adhesion, rapid proliferation, hyphal and extracellular matrix (ECM) formation, and biofilm maturation with subsequent microbial colonization of additional sites (Gulati and Nobile, 2016).

Transcriptomic results showed that MH treatment elicited a pronounced global transcriptional response. Within this broad response, we further identified seven differentially expressed genes (DEGs) associated with biofilm formation (*ALS1, ALS2, ALS4, ALS6, CHT2, ROB1*, and *ECE1*), suggesting that MH may perturb biofilm-related transcriptional programs in *C. albicans* (Chong et al, 2018). The *ALS* gene family encodes cell-surface glycoproteins that mediate adhesion and early biofilm establishment; however, their expression and functional contributions are strongly dependent on species background and environmental conditions (Hoyer et al., 1998; Hoyer and Cota, 2016). In addition, *ALS* genes are significantly upregulated in recurrent vulvovaginal candidiasis, underscoring their importance in host–pathogen interactions (Bonfim-Mendonça et al., 2021). Notably, *ALS6* appears to exhibit context-dependent behavior, as deletion of *ALS6* has been reported to enhance *C. albicans* adhesion to host cells (Zhao et al., 2007), indicating that changes in *ALS6* expression do not necessarily translate linearly into adhesion capacity. Importantly, these transcriptomic signals are consistent with our biofilm phenotypes: MH treatment markedly delayed *C. albicans* biofilm formation, and even at the end of the treatment period, fungal cells failed to establish a compact and mature biofilm architecture. Beyond adhesins, differential expression of *ROB1* (a key regulatory node in biofilm development), *ECE1* (a hypha/biofilm-associated virulence gene), and *CHT2* (involved in cell-wall remodeling) suggests that MH may affect not only initial attachment but also biofilm maturation and structural maintenance. Collectively, MH exhibits an inhibitory/disruptive effect on biofilm formation at both the transcriptomic and phenotypic levels.

In summary, this *in vitro* study demonstrates that MH effectively inhibits the growth and biofilm formation of *C. albicans*. These findings provide an experimental basis for understanding the anti-*Candida* activity of this multi-herb formulation. Further research is necessary to isolate and characterize the specific bioactive compounds responsible for the observed effects and to validate these findings in more complex physiological models, and critically, to conduct systematic safety evaluations, including hemolytic assays *in vitro* and toxicity assessments *in vivo*.

## 5 Conclusion

This study demonstrated that MH exhibits potent broad-spectrum antimicrobial activity against *S. aureus*, *E. coli*, and *C. albicans*, with rapid bacteriostatic and fungistatic effects. Its antibacterial rate against bacteria exceeded 90% within 5 min, while its antifungal rate against fungi surpassed 70% within 10 min. Mechanistic investigations revealed that MH disrupts *C. albicans* biofilm formation by reducing cell surface hydrophobicity, impairing initial adhesion, and altering biofilm architecture. Transcriptomic analysis identified seven differentially expressed genes (ALS1, ALS2, ALS4, ALS6, CHT2, ROB1, and ECE1) associated with biofilm regulation, with RT-qPCR validation confirming significant downregulation of positive biofilm regulators and upregulation of potential anti-adhesive genes under MH treatment.

Based on the *in vitro* findings, the antimicrobial activity of MH may be attributed to the combined effects of its herbal medicines. These herbal medicines could interfere with microbial growth, metabolism, and biofilm formation. The multi- combination of the solution suggests a possibility of multi-target action, which might, in theory, contribute to a lower risk of resistance development compared to single-target agents. However, all assays in this paper were conducted *in vitro*, these interpretations remain preliminary. Further research is necessary to isolate and characterize the bioactive compounds, elucidate the exact mechanisms, and evaluate the safety and efficacy in relevant *in vivo* or clinical settings, such as for vulvovaginal candidiasis.

## Data Availability

The raw data supporting the conclusions of this article will be made available by the authors, without undue reservation.
